# Effect of inoculum size and antibiotics on bacterial traveling bands in a thin microchannel defined by optical adhesive

**DOI:** 10.1038/s41378-021-00309-3

**Published:** 2021-10-22

**Authors:** Yang Liu, Thomas Lehnert, Martin A. M. Gijs

**Affiliations:** Laboratory of Microsystems, Ecole Polytechnique Fédérale de Lausanne, CH-1015 Lausanne, Switzerland

**Keywords:** Engineering, Materials science, Applied optics

## Abstract

Phenotypic diversity in bacterial flagella-induced motility leads to complex collective swimming patterns, appearing as traveling bands with transient locally enhanced cell densities. Traveling bands are known to be a bacterial chemotactic response to self-generated nutrient gradients during growth in resource-limited microenvironments. In this work, we studied different parameters *of Escherichia coli (E. coli)* collective migration, in particular the quantity of bacteria introduced initially in a microfluidic chip (inoculum size) and their exposure to antibiotics (ampicillin, ciprofloxacin, and gentamicin). We developed a hybrid polymer-glass chip with an intermediate optical adhesive layer featuring the microfluidic channel, enabling high-content imaging of the migration dynamics in a single bacterial layer, i.e., bacteria are confined in a quasi-2D space that is fully observable with a high-magnification microscope objective. On-chip bacterial motility and traveling band analysis was performed based on individual bacterial trajectories by means of custom-developed algorithms. Quantifications of swimming speed, tumble bias and effective diffusion properties allowed the assessment of phenotypic heterogeneity, resulting in variations in transient cell density distributions and swimming performance. We found that incubation of isogeneic *E. coli* with different inoculum sizes eventually generated different swimming phenotype distributions. Interestingly, incubation with antimicrobials promoted bacterial chemotaxis in specific cases, despite growth inhibition. Moreover, *E. coli* filamentation in the presence of antibiotics was assessed, and the impact on motility was evaluated. We propose that the observation of traveling bands can be explored as an alternative for fast antimicrobial susceptibility testing.

## Introduction

Bacteria can sense a vast range of environmental signals. Decades of studies on the mechanisms underlying self-propelled oriented bacterial swimming toward preferred niches for colonization, commonly known as chemotaxis, have elucidated pathways of chemosensory signal transduction and response regulation that affect bacterial active motion^1–3^. Most interestingly, motility and chemotaxis play important roles as virulence factors, as these are essential features for pathogens to colonize hosts and to evade their defense mechanisms^4^. For example, the human gastric pathogen *Helicobacter pylori*, which is responsible for chronic gastritis and is associated with gastric and duodenal ulceration, is guided to the mucus lining of the stomach by chemotaxis^5^. An important pathogen in waterborne infections of marine fish, *Vibrio anguillarium*, is directed through mucus-protected skin and intestinal epithelial fish surfaces by components of the mucus that act as chemoattractants^6^. Therefore, from a health care perspective, a further more holistic understanding of bacterial motility, in particular of specific collective and coordinated dynamic patterns of migrating bacterial populations, may open the way to explore new disease-preventive or therapeutic paradigms based on unconventional targets. This perspective is of particular importance with respect to the substantial challenge of fast-spreading antimicrobial resistance (AMR) that the public health system is currently facing^7^. In this context, fast antimicrobial susceptibility testing (AST), which is essential for the provision of the correct antimicrobials, is a key tool to counteract AMR propagation^8^.

Microfluidic high-throughput platforms incorporating chips with (sub)micrometer features, which enable the precise handling and observation of single-cell organisms or small microbial colonies, have helped reveal fundamental aspects of bacterial life, cell-cell interactions and population dynamics^9–11^. Microfluidic chip assays enable accurate imaging and tracking of bacterial populations with single-cell resolution^12^. The so-called “mother machine” developed by Wang et al., comprising an array of parallel dead-end microchannels, is a prominent example with regard to monitoring single bacterial growth and rod-shaped bacterial division on-chip^13^. Using a similar chip design and by averaging the growth rate response to different antibiotics over many individual cells, Baltekin et al. declared a total AST read-out time of less than 30 min^14^. Motility and chemotaxis studies benefit strongly from a versatile microfluidic toolbox, providing controlled and complex on-chip chemical gradient patterns^15^. As a recent example, Lambert et al. engineered a chip featuring an array of 110-μL wells loaded with out-diffusing chemicals to assess the chemotactic behavior of marine microorganism communities^16,17^. In the field of plant biology, Massalha et al. developed a microfluidic device enabling precise dynamic imaging and tracking of root-bacteria interactions^18^. On-chip control of microenvironmental conditions was also used to study microbial taxis with respect to a range of physical parameters, including temperature, magnetic field or light sensitivity^19^.

*Escherichia coli (E. coli)* is a well-characterized model organism for motility studies and uses a run-and-tumble motion strategy by randomly changing the direction of successive straight swimming sections. It exhibits chemotactic behavior for exploring optimal environmental conditions by adjusting the duration of straight runs and/or the frequency of tumbling^3^. In particular, the flagellar motor switches from counterclockwise to clockwise rotation to realize this typical motion pattern. A coordinated protein network controls the sensitivity of ligand-binding chemoreceptors in *E. coli*. The pathway involves the histidine kinase chemotaxis protein CheA and two diffusible response regulators (CheY and CheB). Chemotactic activity is controlled by the phosphorylation level of CheY, mediated by CheA and the phosphatase CheZ. Phosphorylated CheY (CheY-P) triggers clockwise rotation of the flagellar motor by binding to the FliM protein, a constituent of the flagellar motor switch protein complex^3^.

Local depletion of nutrients and oxygen in the culture media during bacterial colony growth is a driving force of bacterial chemotaxis toward other energy resources in the microenvironment. Pioneering work on chemotaxis-induced collective *E. coli* migration patterns was carried out by Adler^20^. This phenomenon appeared as the migration of dense, motile *E. coli* subpopulation(s), so-called traveling bands, when placing a bacterial culture at one end of a capillary tube filled with a chemically defined medium. Self-generated local chemical gradients due to the consumption of nutrients or compounds (galactose and oxygen) are at the origin of these chemotaxis patterns in the tube. Traveling bands have been described mathematically by the Keller-Segel model^21^. However, the latter does not describe the effects of individual variations in swimming behavior, which exist in bacterial populations and were reported first by Spudich and Koshland^22^. Today, microbial cell-to-cell phenotypic heterogeneity that does not have its origin in genotypic differences is well known and has been extensively studied^23,24^. Variability in the expression of motility-related genes is thought to play a role in maintaining chemotactic performance. For instance, Dufour et al. found that nongenetic phenotypic diversity in *E. coli* motility can be controlled by adjusting the expression levels of the CheR and CheB proteins, which regulate the activity of kinase CheA^25^. Recently, Salek et al. used a microfluidic T-maze to expose individual clonal *E. coli* to a sequence of chemosensory decisions, revealing strong heterogeneity in chemotactic sensitivity^26^. Fu et al. revealed that bacteria are able to migrate as a group by sorting themselves and adapting to the chemical gradient steepness in a traveling band despite the continuum of phenotypic diversity^27^. They applied a nongenetic diversity indicator named “tumble bias” (the fraction that tumbling time takes in the duration of an entire swimming segment) to evaluate the individuality in bacterial motility^28^. However, to the best of our knowledge, the effects of inoculum size and antibiotics on the traveling band have not yet been reported.

In this work, we present a study of microbial collective behavior using *E. coli* as a model organism. The impact of inoculum size and antimicrobial action on specific features of bacterial traveling bands will be investigated, which is the first time that the effects of these factors have been reported. We intend to provide new insight into the phenotypic diversity of swimming behavior. The bacterial suspension was confined in a Hele-Shaw microfluidic channel to achieve high-precision imaging of a quasi-2D single bacterial layer. A custom image processing protocol was developed for tracking and quantifying swimming parameters, in particular speed and tumble bias, of coherently migrating bacterial populations at single-cell resolution. We revealed that isogenic bacterial populations growing from different inoculum sizes eventually generated diverse swimming behavior variability. This study was then extended to bacterial populations exposed to different antibiotics. Interestingly, we observed that subminimum inhibitory concentrations (sub-MICs) promoted swimming ability in some cases despite considerable antimicrobial growth inhibition. In situ morphological analysis provided complementary information on *E. coli* filamentation, which is an effect that occurs in the presence of certain antimicrobials. Finally, we propose that the appearance of traveling bands as a function of antibiotic concentration might be an adequate tool for rapid AST approaches.

## Materials and methods

### Materials and chemicals

Norland optical adhesive (NOA68) was purchased from Norland Products (Cranbury, NJ). Sylgard 184 polydimethylsiloxane (PDMS) was acquired from Dow Corning GmbH (Wiesbaden, Germany). Four-inch 550-μm-thick Si wafers and AZ1512 HS (MicroChemicals) photoresist were provided by the EPFL Center of MicroNanoTechnology (Lausanne, Switzerland). The *E. coli ATCC 25922* strain (*E. coli* WDCM 00013 Vitroids^TM^), ciprofloxacin (17850, CAS Number 85721-33-1), ampicillin sodium salt (A0166, CAS Number 69-52-3), gentamicin (G1264, CAS Number 1405-41-0), sterilized mineral oil (BioXtra, M5310, CAS Number 8042-47-5), and trimethylchlorosilane (TMCS, CAS Number 75-77-4) were purchased from Sigma–Aldrich (Buchs, Switzerland).

### Sample preparation

Assays in this work were carried out using the *E. coli ATCC 25922* strain. This organism is a motile and typical AST control strain. We retrieved bacteria from storage at −20 °C and performed overnight bacterial culture in Mueller-Hinton broth (MH) without shaking. Culture without shaking might potentially enhance bacterial motility^29^. Under this condition, the optical density measured at 600 nm (OD600) of the bacterial suspension reached ~1.0 after overnight culture. We diluted the solution to prepare a bacterial suspension with an OD600 value of 0.2 (i.e., 0.5 McFarland standard). Hereafter, two-step dilutions of 200-fold and 10-fold in pure culture medium were used to prepare the bacterial suspension for the antibiotic-related experiments. As quality control experiments, purity, viability and concentration checks prior to each measurement series were carried out by inoculation on a nonselective MH agar plate (after a further 100-fold dilution) and colony counting after overnight culture. Based on 3 serial quality control experiments, the bacterial concentration range for quality control was determined to be (3.1 ± 0.4) × 10^3^ CFU/mL. Correspondingly, the bacterial suspension used for antibiotic-related experiments had a concentration of (3.1 ± 0.4) × 10^5^ CFU/mL, which is in the recommended range for standard AST^30^. We also determined that an OD600 of 1.0 corresponds to 2.7–3.5 × 10^8^ CFU/mL in our study, which is in agreement with a recent systematic optical density and bacterial number study^31^. For the inoculum-size-related experiments, we prepared concentrations of 3 × 10^6^, 3 × 10^5^, 3 × 10^4^, and 3 × 10^3^ CFU/mL by gradual dilution of the bacterial suspension with an OD600 of 0.2. All bacterial suspensions were stored in a small flacon and used within 15 min.

Antimicrobial solutions used in this study were prepared according to the recommended protocols. Ampicillin sodium salt and gentamicin are soluble in DI water. A 1 mol/L hydrogen chloride solution was used for ciprofloxacin. Ciprofloxacin, ampicillin, and gentamicin stock solutions were prepared at concentrations of 25 mg/mL, 50 mg/mL and 36 mg/mL, respectively. Stock solutions were stored at 4 °C and used within 1 week to avoid a risk of declining activity. Prior to the measurements, stock solutions were thermalized at room temperature and gradually diluted in DI water to prepare a series of concentrations according to the potency of each antibiotic (10 times higher than the final target concentrations). Test solution aliquots were prepared by further 10-fold dilution in MH with bacterial inoculation. Target concentrations were chosen according to the MIC range suggested by EUCAST^32^.

### Working principle

**Fig. 1:**
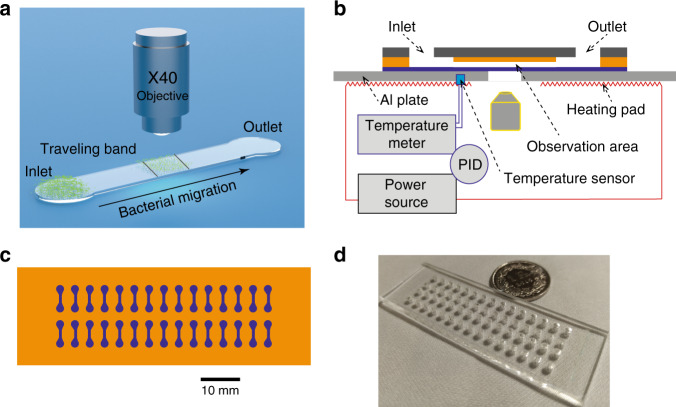
Design and working principle of the chip for quasi-2D confinement of bacterial populations. **a** 3D schematic view of an individual optical adhesive (OA) channel for observing autonomous collective bacterial migration (traveling band) starting from the inlet sample reservoir over several hours of on-chip incubation. **b** Schematic diagram illustrating the imaging configuration and proportional–integral–derivative (PID) temperature control system for on-chip incubation. **c** Top view of the chip with 30 individual OA channels, arranged in two columns. **d** Image of the final chip (75 mm × 25 mm, top view) showing the fluidic access holes in the poly(methyl methacrylate) (PMMA) slide

A schematic illustration of the working principle is shown in Fig. 1a. The experiment was conducted using a simple straight microchannel with a height of a few microns to confine the bacterial suspension. Samples were loaded by pipetting 2 μL of bacterial suspension (inoculum) into the inlet of each microchannel, which was then filled autonomously by capillary flow. Eventually, the channels and inlets were uniformly filled with a bacterial suspension of the same concentration. To prevent evaporation, the inlets and outlets were sealed with sterilized mineral oil (4 μL, BioXtra). The chip was mounted on an Al plate (1.5 mm thick) with a heating pad (MINCO, MN, USA) attached underneath, providing a suitable thermal environment and control for on-chip incubation (Fig. 1b). The heating pad had two parallel rectangular openings (3 mm × 60 mm, for each column of channels) for optical access to the imaging region from below. Assays were carried out at 37 °C. Temperature regulation was based on a proportional–integral–derivative (PID) closed loop using a Lakeshore 336 temperature controller. The chip temperature was probed with a resistance thermometer (RTD) sensor. According to heat transfer simulations (3D model shown in Fig. S1a), a stable temperature distribution can already be achieved after a few minutes with a variation of less than ± 0.5 K over the whole chip area, despite the openings in the heating pad (Fig. S1b, c). The measured temperature at the RTD sensor location stabilized to 37.0 ± 0.1 °C after 3 min (Fig. S1d). In reality, temperature fluctuations and air convection in the ambient space that are not fully rendered in the simulation may impact the temperature stability. Thus, the actual temperature variation over the Al plate surface was 37 ± 1 °C (measured separately with a movable RTD sensor).

Imaging protocols (Fig. 1a, b) were started to record bacterial migration in the format of a traveling band in each individual channel 15 min after sample loading to ensure fully stabilized fluidic and thermal conditions. A top view and a photograph of the whole microfluidic chip are shown in Fig. 1c, d, respectively. The chip features 30 independent straight channels (i.e., 2 columns of 15 channels), allowing simultaneous performance of assays, using either various conditions for assay multiplexing or identical conditions to improve statistical significance. The chip fabrication and imaging procedure will be presented in the following sections.

### Microfluidic chip fabrication

The microfluidic chip was fabricated using an optical adhesive (OA)-based protocol that was adapted from previously published papers^33–35^. Unlike these approaches, our chip consists of a glass-OA-poly(methyl methacrylate) (PMMA) composite structure. We chose Norland NOA68, as this material provides better adhesion to plastics, instead of NOA81, as used by others^36^. For OA microfluidic chip fabrication, a PDMS stamp was required for patterning liquid OA. The PDMS stamp was fabricated using a Si mold that was patterned by standard photolithography (AZ1512 HS resist, thickness 1.6 μm) and by deep reactive ion etching (DRIE). The depth of the Si grooves, corresponding to the final OA channel height, was adjusted by the DRIE duration (etch rate 4 μm/min). The photoresist was removed by oxygen plasma, and a TMCS Si surface treatment was applied prior to pouring PDMS (base-to-curing agent ratio 10:1) on the wafer mold. PDMS was degassed and cured at 80 °C for 2 h. Subsequently, the layer was peeled off from the wafer and cut to form the final stamps.

**Fig. 2:**
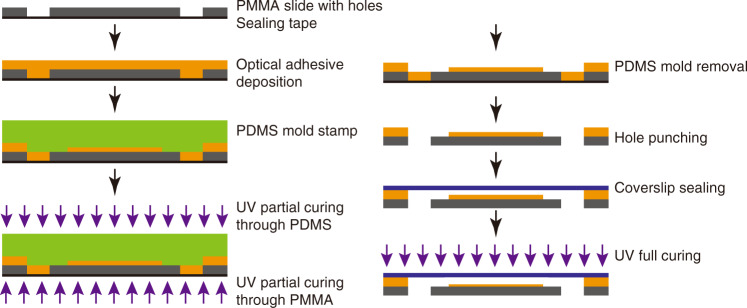
Fabrication process of the shallow microfluidic channels (height 4 μm) defined by imprinting a PDMS stamp into a thin OA layer. A glass coverslip seals the microfluidic channel before complete OA curing

The fabrication steps for the OA microfluidic chip are outlined in Fig. 2. First, liquid access holes to the channels were machined in a PMMA slide (25 mm × 75 mm) by laser cutting. The four edges of the PMMA slide were fixed with tape to facilitate the removal of OA residues on the borders. A layer of liquid OA (thickness ~1 mm) was poured manually on the PMMA slide. Big bubbles could be wiped off using a scalpel. The holes were sealed from the bottom with tape to confine the OA. At this stage, the PDMS stamp featuring the predefined channel structures was aligned and pressed into the liquid OA layer. The OA patterns were solidified by partial curing during UV exposure through the PMMA side (dose 480 mJ/cm^2^) and the PDMS mold side (dose 240 mJ/cm^2^). Subsequently, the PDMS stamp could be removed and reused for the next chip after cleaning with ethanol. Tapes on the edge of the PMMA slide were removed together with eventual overflowing OA, and eventually, the OA-filled holes of the PMMA slide were reopened by punching.

A glass coverslip forms the bottom part of the OA chip and channels. The coverslip was cleaned prior to chip assembly by a protocol of successive soaking steps, comprising (i) acetone, (ii) ethanol, (iii) isopropanol and finally (iv) deionized (DI) water, resulting in a hydrophilic surface with a contact angle of 31 ± 3 degrees (measured by Krüss DSA-30E). Each of these steps was carried out in an ultrasonic bath for 10 min at 45 °C. In addition, the glass coverslip was rinsed with DI water between each step to remove the residual liquid. The cleaned coverslip was brought in contact with the partially cured OA and pressed slightly to seal the chip. Finally, the OA was fully cured by UV light (dose 1.2 J/cm^2^). Eventually, the adhesion of the assembled parts was strong enough to perform liquid manipulations without leakage. Sealing the OA channels with a thin glass coverslip provides a clear advantage for high-resolution imaging using microscope objectives with short working distances.

Each microfluidic channel of the on-chip array had a width of 800 μm and a length of 3 mm. We determined the optimal channel height by testing a range of different OA layer thicknesses (2 μm to 12 μm). Bacteria could not be focused on over the complete height of the channel when the height exceeded 8 μm. However, the bacterial suspensions remained largely confined in the inlet hole if the channel height was too small (<4 μm), eventually resulting in channel clogging. A height of 4 μm was found to be most suitable for the present application (Fig. S2). The observation region is located in the middle of each channel (Fig. 1d). An automated x/y-motor stage was used to scan the array of observation windows for successive imaging of all on-chip assays. For tracking bacterial motion discussed in this work, we used an inverted phase contrast microscope with a 40 × objective (Zeiss LD ACHROPLAN 40 × /0.60 Korr Ph2, working distance 1.8 mm).

### Video recording and image processing

Videos of the bacterial on-chip populations were acquired by a scientific complementary metal-oxide semiconductor (sCMOS) camera pco.panda 4.2 (PCO AG, Germany). Using a high frame rate and resolution (40 fps, 2048 px × 2 048 px), bacterial swimming traces could be precisely captured in the observation areas located at the mid-position of each channel, corresponding to an on-chip field of view (FOV) of 340 μm × 340 μm (Fig. 1b). A video sequence was recorded at 400 frames per 10 s once per hour on each of the observation windows during the whole assay duration (typically 15 h).

**Fig. 3:**
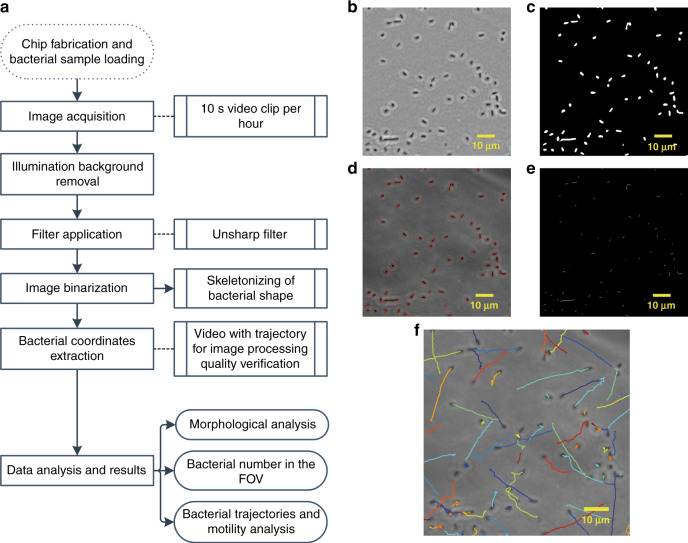
Automated image stack processing and data analysis. **a** Schematic diagram of the workflow for assessing the properties of individual bacteria and bacterial populations in the OA chip. Morphological and motility-related information can be extracted for different experimental conditions and time points based on video analysis. **b** Raw phase contrast image of individual *E. coli* bacteria of a quasi-2D population layer confined in the OA channel (×40 phase contrast objective, FOV ~340 μm × 340 μm, the ImageJ software unsharp filter was applied). (**c**) Binarized images processed by thresholding the image shown in (**b**). **d** Red contours indicate each identified bacterium. (**e**) Image showing the skeleton of each bacterium obtained by replacing the 2D bacterial shape in (**b**) with a 1D line. **f** Bacterial swimming trajectories obtained by attributing an index to each individual bacterium on a consecutive image stack (40 frames, i.e., an observation time of 1 s)

Automated imaging and data processing were performed after collecting the experimental raw data. Image processing was implemented with ImageJ software. As shown in Fig. 3a, the main steps of the protocol are background removal, filtering, image binarization by thresholding, and bacterial boundary detection. A typical sequence of corresponding microscopy images (Fig. 3b–e, zoomed in for details) illustrates the different processing steps. In the raw phase contrast image, bacteria are darker than the background or defects. The first step was to perform illumination background subtraction, which was obtained by taking an image without a chip in the optical path of the microscope. Subsequently, an unsharp filter was applied to the image (radius = 10 pixels, intensity = 0.9) to enhance the contrast between bacteria and background (Fig. 3b). This image is binarized by means of a threshold method (ImageJ “IsoData”), as shown in Fig. 3c. Then, the center position and the boundaries of each individual bacterium were determined and highlighted by red lines (Fig. 3d, using ImageJ “Analyzing particles”). Furthermore, the skeleton of each bacterium could be analyzed by converting the 2-D white geometry in Fig. 3c into a skeletonized 1-D line (Fig. 3e). Occasionally, bacteria may stick together, forming pairs or small clusters.

Datasets of bacterial coordinates were extracted from the processed image stacks. Subsequent data processing was performed by means of MATLAB (R2018b) programs. We used an established tracking algorithm to record the swimming paths of individual bacteria in small on-chip populations and to perform trajectory analysis^37^. An example of a high-resolution image is shown in Fig. 3f, where trajectories of bacteria have been identified with different colors. This image corresponds to a stack of 40 frames, i.e., an observation time of 1 s (zoomed in to a frame of 90 μm × 90 μm in this case). In a time lapse of 1 s, bacterial trajectories mainly correspond to straight or slightly bending segments of motion; only a few microbes tumble frequently on this time scale. Entire trajectories observed in the full FOV (400 frames, recorded over 10 s) indeed show random sequences of straight runs and tumbling (Movie S1). Eventually, we were able to characterize different properties of individual bacteria and their collective migration behavior, including cell body elongation under antimicrobial stress (filamentation), transient bacterial density profiles (traveling bands), and swimming parameters, in particular speed and tumble bias distributions.

### Statistical analysis

All experiments shown in the present work were carried out with 3 replicates on-chip. Statistical analyses of swimming speed, microbial skeleton size, effective diffusion coefficient and tumble bias were performed using MATLAB R2018b and Python (packages: pandas and SciPy). The data related to statistical comparison were tested for normality using the Lilliefors test first and were then analyzed statistically with a *t* test for independent variables if the data were normally distributed. Otherwise, a Mann–Whitney U (MWU) test was used. Significant effects were reported for a *p* value < 0.05. Statistical significance tests were performed using Python (packages: Scipy and statsmodels).

## Results

### Collective migration due to chemotaxis: observation of traveling bands

**Fig. 4:**
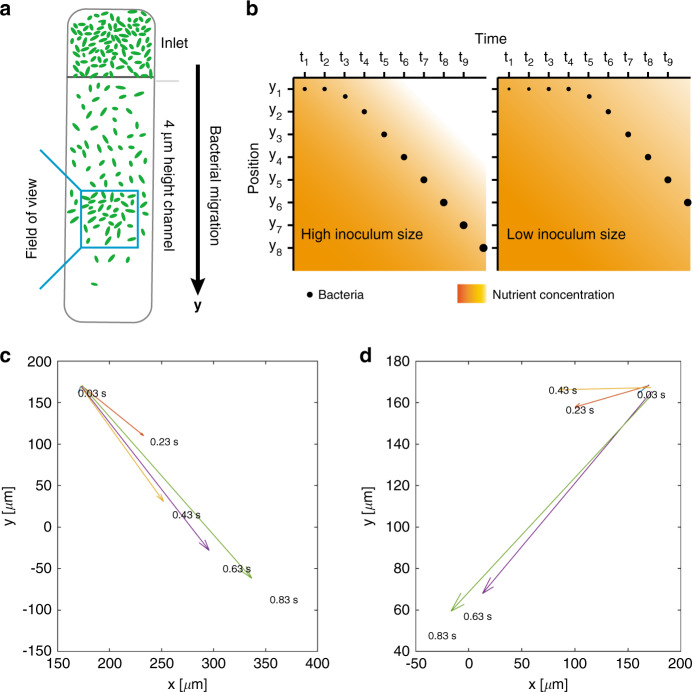
**a** 2D schematic view of bacteria spreading out from the inlet into the OA channel after several hours of on-chip incubation (no flow applied). A bacterial traveling band, *i.e*., a local and transient increase in the bacterial density, may be observed in the FOV. **b** Illustration of the driving force for traveling bands of bacteria with high and low inoculum sizes. Orange color gradients show the nutrient concentration as a function of time and space. The “white” color corresponds to the region in which nutrients are mostly consumed and have a low concentration. The black solid circles indicate the traveling band peak position as a function of time. The size of the circles represents an increasing total number of bacteria in the microfluidic chip due to growth. A larger inoculum size results in faster growth, and thus faster consumption and earlier depletion of nutrients. Therefore, the formation of collective migration for lower inoculum sizes has a time lag compared to the higher inoculum size. **c** The swimming vector calculated by summarizing all vectors of the individual swimming trajectories within the FOV (5 × 10^5^ CFU/mL, Mueller-Hinton broth (MH), observation at 6 h). The vectors were calculated for different time intervals (indicated in the figure). The *y*-component of the vector indicates that the bacterial subpopulation migrates parallel to the channel as a result of chemotaxis. **d** Repetition of the experiment and vector calculation with the same experimental conditions as in (**c**). In contrast to (**c**), the x-component points toward the negative direction, demonstrating the randomness of the lateral collective chemotaxis motion

Coherent migration of bacteria due to chemotaxis through the microchannel appearing as traveling bands could be observed in the FOV (passive migration by diffusion is not relevant in the present case). As shown schematically in Fig. 4a, traveling bands correspond to a transient increase in the microbial density. At the beginning of each experiment, the bacterial density in the microchannel was very low; thus, hardly any bacteria could be observed in the FOV. Considering the typical inoculum size of 3 × 10^5^ CFU/mL used in our assays and the low volume of ~0.36 nL of the channel portion observed, less than 1 bacterium is expected to be found in the FOV. After on-chip incubation for 3 to 6 h, the number of bacteria in the inlet reservoir increased exponentially, resulting in a local depletion of nutrients and other chemical attractants depending on the inoculum size (orange color gradients in Fig. 4b). Higher inoculum size leads to faster nutrient consumption and thus earlier nutrient depletion compared to lower inoculum size. This self-generated chemical gradient due to local nutrient depletion activated chemotaxis, and a subpopulation of more motile bacteria started migrating into the microchannel in a coherent collective manner (black solid circles in Fig. 4b). The resulting traveling bands may be recorded at the FOV location of the chip as transient peaks in the microbial population density. In the shallow geometry of our microfluidic channel, both the morphological information and traveling bands formed in a quasi-2D bacterial layer were observed and analyzed.

As an example, we calculated the overall swimming vectors for different time intervals (Fig. 4c). The vector calculation depended on the choice of the time intervals. Longer time intervals result in larger vectors and a more accurate indication of the migration direction. The *y*-component of the vectors corresponds to migration of the bacterial subpopulation in the FOV parallel to the channel axis, i.e., from the inlet toward the outlet due to self-generated chemical gradient-activated chemotaxis. The vectors also have a nonnegligible x-component, corresponding to lateral swimming patterns. The origin of migration in the x-direction might also be related to self-generated chemotaxis effects enabled by the large width of the channel (800 μm wide). However, this motion may be oriented similarly to either direction perpendicular to the channel orientation. This is exemplified by the vector calculations for two subsequent experiments carried out under comparable conditions. In Fig. 4c, all vectors point to positive x-directions, whereas in Fig. 4d, the lateral chemotaxis direction is oriented to negative x-values. Lateral migration was analyzed for the same time scales in both cases.

### Morphological analysis of bacterial shape

**Fig. 5:**
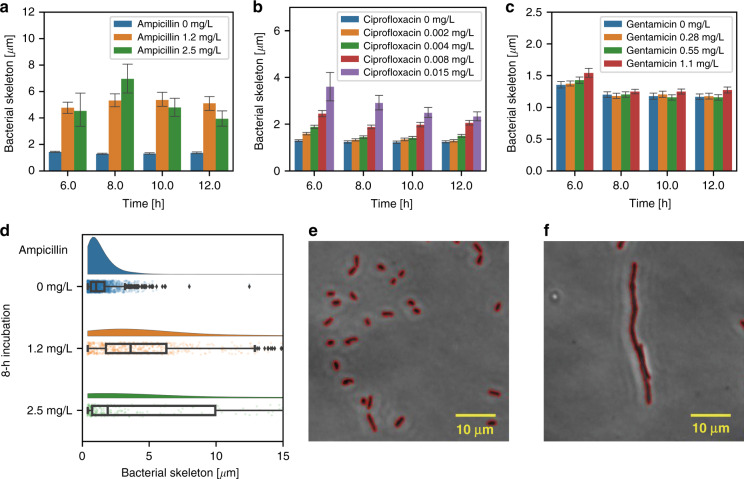
*E. coli* morphological analysis. Mean bacterial skeleton length as a function of incubation time for *E. coli* exposed to ampicillin (**a**), ciprofloxacin (**b**), and gentamicin (**c**) (MH at 37 °C, 3 repetitions of each condition, error bars represent ± 2SE). The bacterial skeleton considerably elongates in the presence of ampicillin. The filamentation effect is less pronounced in the presence of ciprofloxacin and is nearly invisible with gentamicin. **d** Boxplot of the bacterial skeleton length distribution after 8-h on-chip incubation with ampicillin at different concentrations. In the presence of ampicillin, bacterial skeletons show a significantly wider length distribution than under normal growth conditions. **e** *E. coli* cells (indicated by red contours) after 8-h on-chip incubation without antimicrobials. **f** Image and contour of a single elongated *E. coli* bacterium after on-chip incubation with 2.5 mg/L ampicillin for 8 h

In the first stage of our study, we analyzed bacterial morphology on chip by considering a subpopulation in the same FOV of the chip, where traveling bands were also visualized. The analysis considers only the motile fraction of the microbial population of interest in this context, excluding the nonmigrating population that remained in the channel inlet. Figure 5a–c shows the mean skeleton length of *E. coli* obtained automatically by a MATLAB program as a function of on-chip incubation time in MH with three different antibiotics (ampicillin, ciprofloxacin and gentamicin). For normal bacterial growth conditions (MH without antimicrobials), the average cell body length was 1.43 ± 0.04 μm after 6 h (blue bars in Fig. 5a). This value is comparable to those obtained with bacteria incubated in 25-mL flasks (1.47 ± 0.04 μm, *p* value by MWU test: 0.093). We conclude that incubation in the microchannel has no adverse effect on bacterial growth. As a reference for assays involving antibiotics in the present work, we determined the following MIC value ranges from *E. coli* growth curves determined by optical density measurements at 600 nm (OD600): (i) 2.5 < MIC ≤ 5.0 mg/L for ampicillin, (ii) 0.008 < MIC ≤ 0.015 mg/L for ciprofloxacin, and (iii) 1.1 < MIC ≤ 2.2 mg/L for gentamicin (Fig. S3a–c).

Exposure to antimicrobials in the sub-MIC range may have a significant effect on bacterial morphological development, referred to as filamentation. The strongest elongation of the *E. coli* skeleton with respect to normal growth was observed for ampicillin. Figure 4a shows the bacterial size increase and evolution with incubation time for two different ampicillin concentrations (1.2 mg/L orange bars and 2.5 mg/L green bars; the latter concentration is close to the MIC). In both cases, elongation of a factor of 3 to 4 with respect to normal growth was observed. The average size first increased and then decreased due to cell lysis in the later growth phase. This variation over time (6 h to 12 h) was more pronounced for the highest concentration in this case (*i.e*., for 2.5 mg/L ampicillin). The variability of bacterial growth, expressed as the standard error (SE) of the skeleton length (± 2SE or 95% confidence interval), is much smaller under normal growth conditions than for the two ampicillin cases, indicating that antimicrobial stress frequently generates nonuniform bacterial divisions. As shown in Fig. 5b, ciprofloxacin generated a less pronounced filamentation effect than ampicillin. The maximum average skeleton length did not exceed 3.6 ± 0.6 μm (0.015 mg/L at 6 h) in comparison to 7.0 ± 1.2 μm for ampicillin (2.5 mg/L at 8 h, *p* value by MWU test: 0.15). However, in the case of ciprofloxacin, the average bacterial elongation increased gradually for incubation with higher antimicrobial doses and over the whole assay duration (6 h to 12 h). The size uniformity decline was strongest at 0.015 mg/L, i.e., close to the MIC (Fig. 5b, purple bars). A slight decrease in bacterial length with incubation time was also observed. In contrast to the previous findings, bacterial growth in gentamicin did not show a clear filamentation effect (Fig. 5c). Although we observed that the average bacterial size at 1.1 mg/L gentamicin (red bars) was slightly higher than those at the other concentrations, elongation was far less pronounced than those for ampicillin (1.2 mg/L at 6 h, *p* value by MWU test: 3 × 10^−87^) or ciprofloxacin (0.008 mg/L at 6 h, *p* value by MWU test: 10^−29^). Correspondingly, bacterial divisions were more uniform (small SE in Fig. 5c) even for a gentamicin concentration just below the MIC.

A more detailed analysis of the filamentation effect in ampicillin is presented in Fig. 5d, showing the bacterial skeleton length distribution and median values for different ampicillin concentrations after 8-hour on-chip incubation. Figure 5e, f shows images of *E. coli* with highlighted skeletons incubated in MH and an example of a strongly elongated cell in the presence of ampicillin, respectively.

### Analysis of traveling bands: influence of inoculum size and antibiotics

**Fig. 6:**
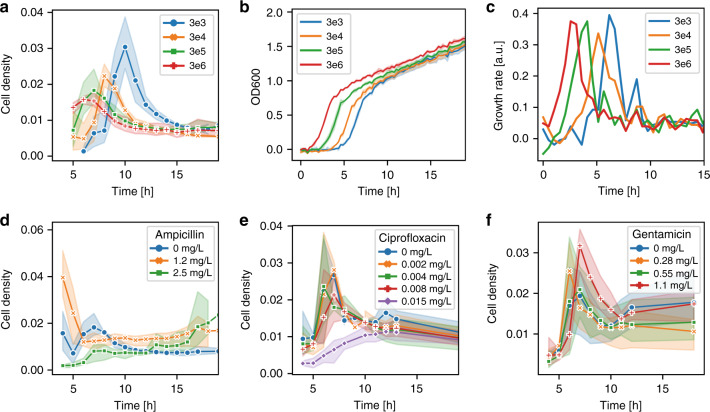
Time-dependent bacterial density profiles (traveling bands). A transient increase in the *E. coli* density is generally observed in the FOV of the microchannel after a few hours of on-chip incubation, corresponding to a collective bacterial migration pattern referred to as the traveling band. **a** Cell density in the FOV as a function of time for bacterial suspensions originating from different inoculum sizes. Cell density peaks appear in the FOV on different time scales. The strongest delay due to the slowest traveling and a stronger cell density increase were observed for the lowest inoculum size. **b** Standard OD600 growth curves obtained on a plate reader (24-well plates). The growth curves indicate that lag phases prolonged with decreasing inoculum size, corresponding to the peak delays observed in (**a**). **c** Growth rate curves (time derivatives of (**b**)) show a corresponding shift of the maximum growth rate. (d-f) Traveling bands for *E. coli* subjected to different antimicrobial conditions, i.e., ampicillin (**d**), ciprofloxacin (**e**), and gentamicin (**f**). For concentrations below the MIC, traveling bands were observed in all cases. For concentrations approaching the MIC, bacteria could still be observed in the FOV for ampicillin (green curve, 2.5 mg/L) and ciprofloxacin (purple curve, 0.015 mg/L), but no clear transient traveling bands occurred. For gentamicin at the concentration tested, which was closest to the MIC (red curve, 1.1 mg/L), a clear traveling band with the highest peak amplitude occurred. No bacteria were detected in the channel at the next higher concentration tested (2.2 mg/L ≥ MIC). The presence or absence of traveling bands is related to growth/motility inhibition and may be explored as an indicator for *E. coli* MIC values. Inoculum concentrations are labeled as 3en, corresponding to 3 × 10^n^ CFU/mL. The error bars (or bands) correspond to ± 2SE

We then studied the effect of the *E. coli* inoculum size on the traveling band peak intensity and the peak delay under no-drug culture conditions in pure MH (Fig. 6a, inoculum range from 3 × 10^3^ CFU/mL to 3 × 10^6^ CFU/mL). Given the sample volume of 2 µL introduced in the channel inlet reservoir, for a concentration of 3 × 10^3^ CFU/mL, only ~6 bacteria are expected to be initially present in the microchannel. The transient cell density peak had the highest amplitude and showed the longest delay (10 h) for the smallest inoculum size (3 × 10^3^ CFU/mL, blue curve in Fig. 6a). In Fig. 6a (as well as in Fig. 6d–f), cell density is defined as the fraction of the total bacterial body area to the area of the FOV, reflecting more accurately the actual biomass. Due to possible filamentation in the presence of antibiotics, the corresponding bacterial number densities (Fig. S4a) may be smaller and not proportional to the integrated cell body area obtained by image analysis, especially in the case of ampicillin (see Figs. 6d and S4b). In Fig. 6a, delays and peak amplitudes decreased with increasing inoculum size. As nutrient depletion (Fig. 4b) in the inlet generated by exponentially growing bacterial populations is at the origin of collective chemotactic behavior, the traveling band delay should in principle be related to bacterial growth. A higher number of bacteria means faster depletion in the inlet, leading to earlier bacterial migration out of the inlet in the form of a traveling band. OD600 growth curves measured in a well plate showed different lag phases, indicating essentially a right-hand shift for decreasing inoculum size, whereas the overall curve shape was conserved (Fig. 6b). Correspondingly, the maximum growth rate appeared to be shifted on the time scale according to lag phase extension (Fig. 6c, curves are time derivatives of Fig. 6b). However, the lag phase difference cannot explain the pronounced cell density peak amplitude variation observed in Fig. 6a. Actually, according to the OD600 curves, cell densities are expected to be very similar at the time when the peak for the corresponding inoculum size is observed in the FOV (i.e., ~1.0 OD600 in Fig. 6b).

Traveling bands generated by bacterial populations incubated with antibiotics are the focus of the second part of our study (Fig. 6d–f). In the presence of antibiotics, features of traveling bands depend on both growth inhibition due to the antimicrobials and possible alterations in bacterial mobility (for example, when bacteria become more elongated; for the higher doses of ampicillin and ciprofloxacin, one can expect a reduced mobility). At sub-MIC values (according to the MIC reference values in Fig. S3), traveling bands were observed in the FOV for the three antibiotics tested (ampicillin, ciprofloxacin and gentamicin), whereas no bacteria were detected above the MIC (for *t* ≤ 19 h, tested for ampicillin 5.0 mg/L, ciprofloxacin 0.030 mg/L, and gentamicin 2.2 mg/L, the results not shown). In the concentration range close to the MIC, in particular for 2.5 mg/L ampicillin and 0.015 mg/L ciprofloxacin, enhanced growth inhibition monitored by prolonged lag phases and filamentation effects dominated (Fig. S3a, b and Fig. 5a, b, respectively). No dense traveling bands occurred in these cases; however, a slight bacterial population density variation could still be observed, mainly because occasionally migrated bacteria continued to grow in the channel (green curve in Fig. 6d and purple curve in Fig. 6e, respectively).

For these two antibiotics, we can therefore determine a lower limit for the *E. coli* MIC value range by identifying the concentration where clear traveling bands no longer appear. These values are consistent with the MIC ranges obtained by OD600 growth curves (Fig. S3a, b). The situation is different in the case of gentamicin (Fig. 6f), where traveling bands appeared for all concentrations shown, including those that featured the strongest lag phase extensions (Fig. S3c, green and red curves, i.e., from ~ 2 h in pure MH to ~7 h at 0.55 mg/L and ~ 13 h at 1.1 mg/L) or corresponding growth rate peak shifts (Fig. S3f, green and red curve). The transient peak amplitude was even highest for the concentration that was closest to the MIC (i.e., for 1.1 mg/L, red curve in Fig. 6f). No growth occurred at the next higher concentration tested (2.2 mg/L). Interestingly, no pronounced or systematic variation in the delay of the traveling band peaks at sub-MIC concentrations were observed. Extension of the lag phases for increasing antimicrobial concentrations, as monitored by the OD600 bacterial growth curves (Fig. S3), did not seem to have a corresponding effect on the transient cell density curves. The peak value for 1.2 mg/L ampicillin appeared at ~4 h (orange curve in Fig. 6d, no bacteria are visible in the channel at *t* ≤ 3 h), which was earlier than for normal growth conditions (blue curve in Fig. 6d), even though the growth rate peaks appeared at the same time (Fig. S3d), indicating that growth in the microchannel does not dominate the traveling band dynamics.

For ciprofloxacin, all traveling band peaks appeared nearly at the same time, although the growth rate curves showed a clear time shift for 0.008 mg/L (Fig. 6e and red curve in Fig. S3e). This discrepancy was even more pronounced for gentamicin. As mentioned above, OD600 growth curves for 0.55 and 1.1 mg/L indicated that growth under these conditions was not fully inhibited but occurred with a strong delay. However, there was no corresponding time shift in the traveling bands for this concentration range (Fig. 6f). Unlike the assays testing different inoculum sizes (Fig. 6a), where the traveling peak positions shifted according to lag phase extension (Fig. 6b), such a tendency was not observed for incubation with antimicrobials. As will be discussed below, exposure to antimicrobials might enhance the effect of collective migration. Moreover, *E. coli* did not show significant filamentation for gentamicin in the sub-MIC concentration range (Fig. 5c), in contrast to ampicillin and ciprofloxacin, which may have an impact on the different dynamic migration patterns generated in the presence of the different antibiotics.

### Motility analysis for different inoculum sizes

To elucidate the effects of morphology and swimming behavior changes on the traveling bands, we performed a detailed bacterial motility analysis of migrating *E. coli* populations. The analysis is performed from several perspectives, namely, the swimming speed and the tumble bias (TB) distribution, and the resulting effective microbial population diffusion properties. The motility analysis is based on individual swimming trajectories of the bacterial subpopulation appearing in the FOV at a given point in time, particularly at the peak of the transient cell density of the traveling band. A MATLAB particle tracking algorithm was applied to identify specific bacteria and to calculate the set of individual trajectories (Movie S1)^37^. Only trajectories longer than 1 s (frequency ~80%) were used to improve the reliability of the calculation. Once individual bacterial trajectories are identified, the swimming speed *v*_*0*_ is calculated as the total length of the linear segments in a specific trajectory divided by the migration duration (including time spent for tumbling). The mean speed *v*_*mean*_ is the average of the *v*_*0*_ values of all trajectories that appeared in the videoclip.

**Fig. 7:**
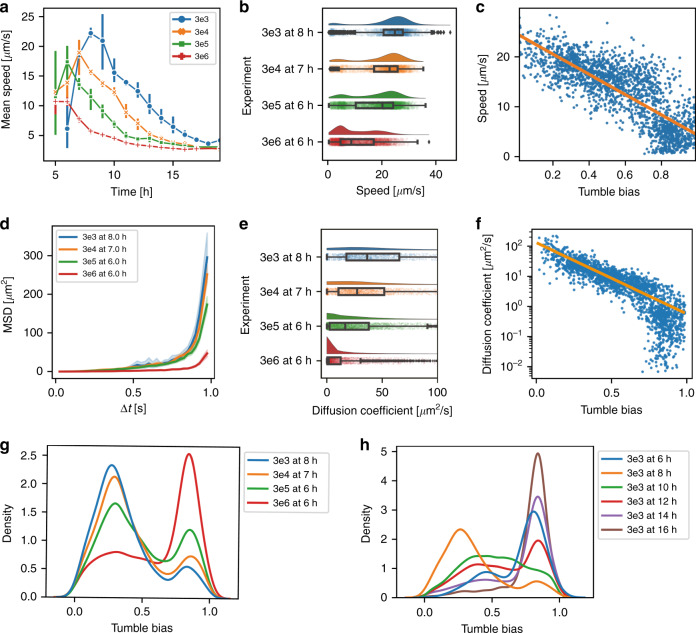
Bacterial motility analysis for different inoculum sizes. **a** Mean swimming speed *v*_*mean*_ in the FOV as a function of time, derived from individual bacterial trajectories. *v*_*mean*_ peak delays and peak values increased as the inoculum concentration decreased. The *v*_*mean*_ curves show the same trend as the cell density curves in Fig. 6a. **b** Speed *v*_*0*_ distribution of all bacteria in the FOV for the time point when the *v*_*mean*_ peak occurred. Two subsequent or overlapping Gaussian distributions, depending on inoculum size, can be observed. This implies the existence of two distinct bacterial subpopulations with lower and higher swimming speed ranges. **c** Swimming speed *v*_*0*_ vs. tumble bias (TB); each dot represents the data of one trajectory. *v*_*0*_ is inversely proportional to TB. **d** *MSD* as a function of a time interval Δ*t* for evaluating the effective bacterial diffusion properties at the time point of the highest *v*_*mean*_ for the corresponding inoculum size. Active swimming results in a strong deviation from the linear *MSD*(Δ*t*) relation expected for Brownian motion. **e** Distribution of the effective diffusion coefficient *D*_*eff*_. *D*_*eff*_ decreases for increasing inoculum size. The *y*-scale indicates the density of individual *D*_*eff*_ values (arbitrary scale). **f** Effective diffusion coefficient *D*_*eff*_ vs. TB. *D*_*eff*_ is inversely proportional to TB. **g** TB distribution showing two peaks as well. The high TB population density rises, whereas the low TB population decreases for higher inoculum sizes. Distinct TB ranges directly reflect different bacterial phenotypes. **h** TB distribution for 3 × 10^3^ CFU/mL inoculum size observed in the FOV at different time points. High-TB subpopulations appear at later stages. Inoculum concentrations are labeled as 3en, corresponding to 3 × 10^n^ CFU/mL. The error bars (or bands) correspond to ± 2SE

As shown in Fig. 7a, the mean speed *v*_*mean*_, peak delay and peak amplitude all increased with decreasing inoculum size (the behavior of the latter two parameters is in agreement with the cell density plot of Fig. 6a). The motility analysis based on individual trajectories (Fig. 7a) was compared with a conventional pixel change analysis. We considered the pixel changes in two consecutive binarized image frames (Fig. S5). More pixel changes (normalized with respect to the total area of bacteria in the FOV) indicate a higher overall bacterial motility. The latter analysis is relatively simple but does not reveal individual bacterial trajectory properties. However, the overall shape and the peak appearance of the curves obtained with both methods correspond well. Figure 7b shows the *v*_*0*_ distribution of individual bacteria (the density of each value is plotted on the y-scale). In all cases, two more or less apparent Gaussian distribution profiles could be distinguished, which implies the existence of two distinct bacterial groups traveling at lower and higher speeds, respectively. For inoculum sizes in the range from 3 × 10^3^ CFU/mL to 3 × 10^5^ CFU/mL, the high-speed group dominates, with its peak position shifting to lower values, whereas the density of the low-speed subpopulation tends to increase slightly. For the highest inoculum size (3 × 10^6^ CFU/mL), the low-speed group was clearly more pronounced than the high-speed group. This evolution of the *v*_*0*_ distribution profiles results in decreasing median speed values for increasing inoculum size, as shown in Fig. 7b, or a lower *v*_*mean*_ peak value for larger inoculum size, as shown in Fig. 7a. These results indicate that a more vigorous swimming behavior is observed at the peak of the traveling band in the FOV for bacteria that were initially less affected by food depletion, forming a population—with their progeny—that therefore took the longest time to arrive at the FOV.

**Fig. 8:**
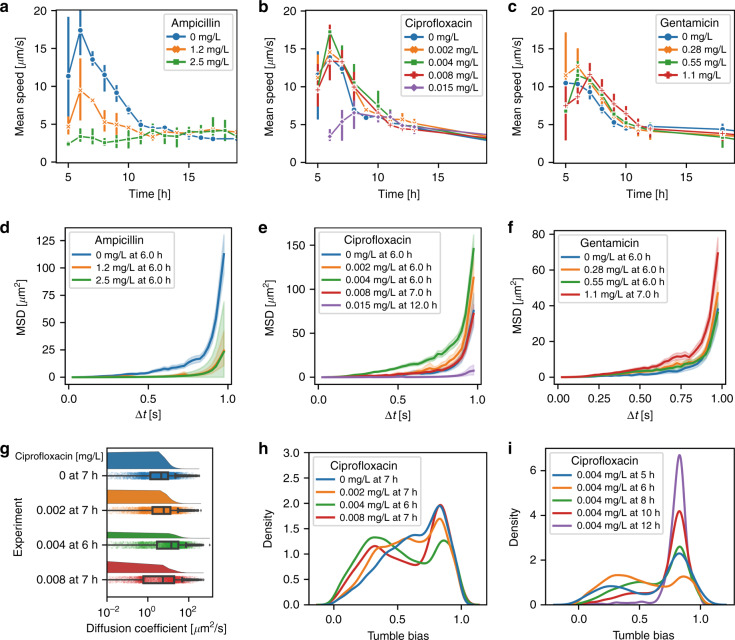
Motility and TB analysis for bacteria in the FOV with different antibiotics (inoculum size 3 × 10^5^ CFU/mL). Mean swimming speed *v*_*mean*_ observed in the on-chip FOV as a function of time for bacteria incubated with ampicillin (**a**), ciprofloxacin (**b**), and gentamicin (**c**). Whereas at sub-MIC, antimicrobials inhibited bacterial growth and generated strongly elongated lag phases (Fig. S3), the swimming ability of bacteria was impeded for ampicillin and ciprofloxacin, while it did not significantly change for gentamicin. Note that the latter antibiotic was the only one that did not result in bacterial elongation.*MSD*(Δ*t*) for the time points of the corresponding *v*_*mean*_ peaks for ampicillin (**d**), ciprofloxacin (**e**), and gentamicin (**f**). In the case of ampicillin, the *MSD* dropped strongly for both concentrations tested. For 0.004 mg/L ciprofloxacin (Fig. 8e, green curve) and for 1.1 mg/L gentamicin (Fig. 8f, red curve), effective diffusion was enhanced. In ciprofloxacin, as for ampicillin, the *MSD* is strongly reduced for concentrations approaching the MIC. **g** *D*_*eff*_ for different ciprofloxacin concentrations. The highest *D*_*eff*_ median value was found for bacteria incubated with 0.004 mg/L ciprofloxacin. **h** Bacterial TB distribution for ciprofloxacin. Two TB subpopulations (low TB and high TB) could be identified on all graphs. The distribution does not strongly depend on the concentration, except for 0.004 mg/L, where the high TB peak is reduced, yielding a more balanced distribution. **i** TB distribution as a function of time for bacteria in 0.004 mg/L ciprofloxacin. The rising high-TB cell fraction contributes to the decreasing mean speed, as shown by the green curve in Fig. 8b, and populations with a high TB evidently need a longer time to arrive within the FOV. The error bars (bands) correspond to ±2SE

The effective diffusion properties of an *E. coli* population in the FOV may be assessed by considering the mean square displacement (*MSD*) as a function of a time interval Δ*t*.

$$MSD\left( {{\Delta}t} \right) = \frac{1}{N}\mathop {\sum }\limits_{i = 1}^N \left( {\left( {x\left( {t_i + {\Delta}t} \right) - x\left( {t_i} \right)} \right)^2 + \left( {y\left( {t_i + {\Delta}t} \right) - y\left( {t_i} \right)} \right)^2} \right)$$

where *t*_*i*_ represents the time lapse for each frame *i* of the image sequence, and Δ*t* is the time interval taken into consideration. In the case of merely passive diffusion due to random Brownian motion, *MSD* would be proportional to the interval time Δ*t*. However, bacteria are active swimmers, and the *MSD*(Δ*t*) relation therefore deviates from the linear relationship. This is emphasized in Fig. 7d, where a strong parabolic-like increase is observed for Δ*t* values approaching 1.0 s for all inoculum sizes investigated. *MSD* data analysis, as well as the data in Fig. 8d–f, corresponds to the time point when the peak *v*_*mean*_ of the corresponding traveling band appeared in the FOV (as observed in Fig. 6a). Obviously, the increase in *MSD* of bacteria originating from a lower initial inoculum size (e.g., 3 × 10^3^ CFU/mL, blue curve in Fig. 7d) was much stronger than that of bacteria cultured with a higher inoculum size (e.g., 3 × 10^6^ CFU/mL, red curve).

An effective diffusion coefficient can be obtained based on the method proposed by Dufour et al.^25^. It is defined as *D*_*eff*_ = *v*_*0*_^2^*τ*/*d*, where *v*_*0*_ is the speed of a bacterium, *d* is the number of dimensions (*d* = 2 for the quasi-2D bacteria confinement in the microchannel), and *τ* is the time scale of the cell directional persistence. The latter is a function of the cell tumble bias, mean tumble angle, and rotational diffusion. We calculated this parameter using a program provided by Dufour et al.^25^ Fig. 7e shows the *D*_*eff*_ distribution for all analyzed bacterial trajectories and corresponding median values. The diffusion coefficient of a nonmotile bacterium subjected to Brownian motion may be estimated to be 0.45 μm^2^/s^38^. The *D*_*eff*_ median values derived here are higher than those for passive diffusion for all inoculum sizes, with a clear trend of increasing *D*_*eff*_ for bacteria cultured from lower inoculum sizes.

A tumble event in the *E. coli* motility pattern corresponds to a sudden change in swimming direction, and TB is defined as the probability that a cell is tumbling^28^. Examples of the trajectories with different TBs are shown in Fig. S6. TB is inversely proportional to *v*_*0*_ and *D*_*eff*_ (Fig. 7c and f). For instance, a low inoculum size with a higher density of low-TB cells thus results in a higher *v*_*mean*_. TB distributions are represented as probability density functions obtained by kernel density estimation. By assigning tumble events and straight runs to each bacterial trajectory, the TB distributions for different inoculum sizes were obtained (Fig. 7g). Analysis based on the corresponding 10-s video clips shows a wide distribution of TB values over the whole range from 0 to 1.0. The TB distribution in general shows two subpopulations, i.e., a low-TB group centered at ~0.25 and a high-TB group centered at ~0.80. The relative cell densities of the low-TB and high-TB groups varied with inoculum size. For smaller inoculum sizes, i.e., 3 × 10^3^ CFU/mL and 3 × 10^4^ CFU/mL (blue and orange curves in Fig. 7g, respectively), low TB values dominated. As the inoculum size increased, TB values shifted to the higher range, with a more balanced distribution of 3 ×10^5^ CFU/mL (green curve in Fig. 7g) and a clearly dominating high-TB population of 3 ×10^6^ CFU/mL (red curve in Fig. 7g). Figure 7h displays the TB distribution for an inoculum size of 3 ×10^3^ CFU/mL at successive time points, i.e., for bacteria progressively migrating through the FOV. As the FOV position is fixed, the graphs in Fig. 7g may also be interpreted as the variation of the spatial distribution of TB subpopulations in the microchannel, i.e., over the entire traveling band. Before the arrival of the traveling band peak, high TB values preponderated (at *t* = 6 h, blue curve in Fig. 7g). At the traveling band peak time point, low-TB value populations dominated (at *t* = 8 h, orange curve). Bacteria arriving after the transient peak again featured mainly high TB values (at *t* = 14 h or 16 h, purple and brown curves, respectively). These results reveal that an initially uniform bacterial culture self-organized itself along the traveling band into subpopulations with different TB. As bacterial inoculums were prepared from the same overnight culture, we may assume that all bacteria had the same genotype. The peak amplitude variation therefore implies that an isogeneic population generates subpopulations with different chemotactic behaviors.

### Motility analysis for different antibiotics

We applied the protocols described for motility analysis under normal culture in pure MH to bacterial populations incubated with different antimicrobials. The results for *E. coli* exposed to ampicillin, ciprofloxacin and gentamicin at different concentrations are summarized in Fig. 8 (inoculum size 3 ×10^5^ CFU/mL). The three tested antibiotics affected the *v*_*mean*_ distribution in different ways. Over the assay duration (up to ~12 h), *v*_*mean*_ was significantly lower than for normal growth conditions when subjected to 1.2 mg/L and 2.5 mg/L ampicillin (Fig. 8a, blue and orange curves, respectively). Interestingly, for ciprofloxacin (Fig. 8b) and gentamicin (Fig. 8c), at sub-MIC values, *v*_*mean*_ was roughly maintained at a comparable level for all conditions. A closer look at the graphs, however, revealed that *v*_*mean*_ with ciprofloxacin at 0.004 mg/L was enhanced with respect to normal conditions (Fig. 8b, green and blue curves, respectively). In gentamicin, an overall increase in the *v*_*mean*_ peak was observed for all sub-MIC values tested (Fig. 8c, curves for 0.28, 0.55 and 1.1 mg/L with respect to the blue curve). For concentration values approaching the MIC, *v*_*mean*_ was strongly reduced in the case of ampicillin (Fig. 8a, 2.5 mg/L, green curve) and ciprofloxacin (Fig. 8b, 0.015 mg/L, purple curve). *E. coli* growth was strongly delayed but not completely inhibited at 1.1 mg/L gentamicin (Fig. S3c, red curve), which apparently did not have an impact on the speed distribution. The evaluation of motility for the three antibiotics tested based on pixel change analysis is shown in Fig. S7a–c.

The impact of antimicrobial exposure on the effective diffusion properties of *E. coli* was first addressed by analyzing *MSD* (as explained for Fig. 7d for culture in pure MH). In the case of ampicillin, *MSD* (as well as *v*_*mean*_, Fig. 8a) dropped strongly for both concentrations tested (1.2 mg/L and 2.5 mg/L, Fig. 8d, orange and green curves, respectively). Interestingly, in the presence of ciprofloxacin (Fig. 8e) and gentamicin (Fig. 8f), the *MSD* may be enhanced in some cases with respect to normal culture conditions. Even if the *MSD* remained close to the normal *MSD(*Δ*t)* curve for most concentrations (Fig. 8e, f, blue curves), for 0.004 mg/L ciprofloxacin (Fig. 8e, green curve) and for 1.1 mg/L gentamicin (Fig. 8f, red curve), the effective diffusion was enhanced. For ciprofloxacin, the *MSD* was strongly reduced at concentrations approaching the MIC (0.015 mg/L, Fig. 8e, purple curve).

Overall, these antimicrobial motility assays revealed similar impacts on *v*_*mean*_ and *MSD*. As an example, we discuss the *D*_*eff*_ and TB distributions for ciprofloxacin for different parameters (Fig. 8g–i). *E. coli* in ciprofloxacin at 0.004 mg/L (Fig. 8g, green graph) showed a higher *D*_*eff*_ than at other concentrations, including normal culture conditions (Fig. 8g, blue graph). To further analyze this observation, the corresponding TB distributions are shown in Fig. 8h. The TB distribution indicates a balance between low-TB and high-TB cells for 0.004 mg/L ciprofloxacin (Fig. 8h, green curve), whereas for other concentrations, the high-TB subpopulation was dominant. Eventually, according to the previous findings, this particular fact may generate higher *D*_*eff*_ and *v*_*mean*_. Figure 8i shows the time evolution of the TB distribution for 0.004 mg/L ciprofloxacin. When compared to Fig. 7g for normal conditions, bacterial self-sorting in the traveling band revealed high TB cell ratios before and after the *v*_*mean*_ peak in the FOV and a more balanced distribution at the time when the peak appeared (Fig. 8i, *t* = 6 h, orange curve). Figure S7d, show *D*_*eff*_ reduction for 1.2 mg/L ampicillin and *D*_*eff*_ enhancement for 0.28 mg/L and 1.1 mg/L gentamicin, respectively. The corresponding TB distributions indicate a clear impact in the case of ampicillin (Fig. S7e) and a much less pronounced effect in the case of gentamicin (Fig. S7h). Correspondingly, Fig. S7f, i show the time evolutions of the TB distribution for 1.2 mg/L ampicillin and 1.1 mg/L gentamicin, respectively.

## Discussion

Improvement of 3D particle tracking based on specific functionalities of modern microscopes^39–41^ or advanced algorithms^42^ has extended the possibility to study bacterial or small-molecule dynamics in more complex environments. Nevertheless, these methods are still limited to a low number of moving objects. Moreover, submicron on-chip structures or constrictions enable detailed bacterial imaging with single-cell resolution^43^. By confining bacterial populations in the quasi-2D space of a 4-μm-high microchannel, we were able to perform high-precision tracking for motility analysis and to visualize morphological details based on conventional microscopy, even for relatively high bacterial concentrations. In particular, we observed correlated migration properties that depend on the degrees of phenotypic diversity occurring under different on-chip culture conditions.

We chose a composite microchip design that features an OA channel as the core element. Unlike previously reported methods that use fully NOA81-based microfluidic chips^33–35^, here we provide an alternative way to use NOA68 to fabricate a chip featuring a sandwich structure with multiple materials. The chip provides an overall rigid and accurately defined microfluidic structure that is easier to fabricate than a full glass or polymer chip that might require additional etching or hot embossing techniques^44^. Fluidic access holes and threads for connectors could be readily machined in the PMMA top plate, whereas the bottom coverslip of the chip enables high-resolution imaging of bacteria on an optical microscope. Using OA as a functional intermediate layer, into which microchannels can be directly imprinted, provides a good alternative to other assembly methods (e.g., glass-to-glass bonding), as it serves simultaneously as an adhesion layer for leakage-free sealing of the chip. OA-based chips also have several advantages over common PDMS microfluidic chips^45,46^. The elastic properties of PDMS, which are widely exploited in the design of on-chip valves and pumps^47^, may be a challenge for the present application, particularly for controlling residual flow in the microchannel. In our experiments using the rigid OA-based structure, we observed that residual flow instantaneously stopped once capillary filling was finished. An advantage of PDMS is that micro- and nanostructures can be accurately replicated from molds created by optical lithography in SU-8 or on Si wafers. We used this feature to precisely define the μm-size height of the OA channel by imprinting with a PDMS stamp. An additional advantage of OA-based chips is the low level of autofluorescence (~4 times lower than PDMS), a feature that is important for assays involving fluorescently labeled organisms and molecules^35^. PDMS is gas-permeable, which is a useful property in certain biological assays. However, oxygen permeability may bring about problems for certain on-chip chemical synthesis applications, which would make OA chips a more suitable choice^48^.

Subsequently, we analyzed bacterial traveling bands and individual swimming trajectories. A bacterial traveling band may be described using the classical Keller-Segel (K-S) equations^21^ and an extended model based on the following equations^49^.

1 $$\frac{{\partial b}}{{\partial t}} = \nabla \cdot \left( {\mu \left( s \right)\nabla b} \right) - \nabla \cdot \left( {\chi \left( s \right)b\nabla s} \right) + g\left( {b,s} \right) - h\left( {b,s} \right)$$

2 $$\frac{{\partial s}}{{\partial t}} = D_{chem}\nabla ^2s - f\left( {b,s} \right)$$

where *b(x,t)* is the bacterial cell density at position *x* and time *t*, *s(x,t)* is the chemical attractant concentration, *μ(s)* is the bacterial diffusion coefficient due to Brownian motion, *χ(s)* is the chemotactic coefficient related to active swimming, *g(b,s)* and *h(b,s)* are functions describing cell growth and death, respectively, *f(b,s)* is a function describing attractant degradation, and *D*_*chem*_ is the diffusion coefficient of the chemoattractant. Bacterial swimming is thus described by two components, i.e., passive and chemotactic diffusion, respectively. However, the swimming heterogeneities existing in a bacterial population are not reflected directly by these two equations. Fu et al. established a relationship between *χ(s)* and TB, saying that *χ(s)* increases as TB decreases^27,28,50^. In this way, the phenotypic diversity of swimming motility is represented by TB and can be implemented in the K-S model.

Developing and maintaining phenotypic diversity can be a bet-hedging strategy in a bacterial population to ensure survival in challenging environments^51^. Nongenetic heterogeneity may also impact the coordinated collective behavior of bacterial populations, with a higher degree of diversity usually implying less coordination. In our work, we observed a clear impact of inoculum size on bacterial collective migration appearing as traveling bands. A smaller inoculum size generated an enhanced coordination of migration properties, resulting in traveling bands with high cell density (e.g., Fig. 6a blue curve, inoculum size 3×10^3^ CFU/mL). Expression of phenotypic diversity was found in the variation of the relative densities of high-TB and low-TB subpopulations for different inoculum sizes (Fig. 7g), which are correlated to the corresponding *v*_0_ speed distributions (Fig. 7b). For instance, straight swimming segments (runs) dominated when TB was low, resulting in an enhanced density of bacteria with higher swimming speeds (e.g., blue curves in Fig. 7b and g, inoculum size 3 × 10^3^ CFU/mL). Likewise, a low median speed value was observed when high-TB populations dominated (red curves in Fig. 7b and g, inoculum size 3×10^6^ CFU/mL). Referring to Eq. 1, low TB corresponds to a high chemotactic coefficient *χ(s)* and thus increases the effective diffusion expressed by *D*_*eff*_ in our work (Fig. 7e and f), which can be understood as the sum of the chemotactic coefficient *χ*(s) and the passive diffusion coefficient *μ(s)* appearing in the K-S equations. Looking at the spatial distribution in a traveling band (deduced from observation at successive time points in the FOV), we noticed that the high-TB cells appeared at higher concentrations at a later stage; thus, they migrated preferentially in the tail of the band due to bacterial self-organization (Fig. 7h).

Phenotypic diversity, reflected by the TB distributions in the present study, originates from variations in cellular gene expression and protein content^52^. The protein content inherited by daughter cells strongly depends on the protein content of the mother cells. For instance, it has been demonstrated that exponential-phase *Bacillus subtilis* cultures have a mixture of motile and nonmotile cell types, depending on whether the transcription factor for motility is taking effect^53^. This heterogeneity, manifested by bifurcation into distinct subpopulations, usually refers to bistability. Bistability was also demonstrated in *E. coli* persister cells, which may grow at different rates or react to antimicrobial exposure in different manners^54^. Bistability occurring in bacterial populations originates in unimodal noise in gene expression, such as random fluctuations in the rates of synthesis and degradation of the cognate gene product. The existence of low-TB and high-TB microbial subpopulations analyzed in our study can also be considered a bistability phenomenon. It seems that bacteria are able to sense the initial inoculum size as one of the noise sources in gene expression to adopt a strategy to promote a specific TB distribution during cell culture, e.g., the low-TB cell population in the case of small inoculum size (Fig. 7g). Eventually, different inoculum sizes induced different swimming phenotypic variabilities. The underlying mechanisms at the molecular regulation level need further investigation.

Our antimicrobial assays were based on compounds with different functional mechanisms, namely, ampicillin, ciprofloxacin and gentamicin^55^. Ampicillin is a typical β-lactam antimicrobial that inhibits cell wall synthesis. Ciprofloxacin targets topoisomerase II (DNA gyrase), thus inhibiting the duplication of DNA. Gentamicin impedes protein synthesis by binding to the A site of 16 S ribosomal RNA. *E. coli* tends to elongate upon β-lactam antimicrobial exposure and culture, an effect known as filamentation, followed by cell lysis^56^. Genotoxic antibiotics, such as ciprofloxacin, inducing the SOS response, which refers to a DNA repair network, may change the *E. coli* rod shape into multichromosome-containing filaments^57^. In our morphology study, we observed different degrees and forms of *E. coli* filamentation, reflecting different effects of antimicrobial stress in the cytoplasm or on the cell wall (Fig. 5). Morphological differences may also be one of the sources leading to different swimming behaviors. However, no clear modes of action show that these antimicrobials directly influence the protein synthesis of the flagellar motor.

Pathogen virulence is often linked to bacterial motility^4^. Flagellar motility was shown to be important in the process of effective infection^58,59^. Our in vitro study provides new insights into the motility change upon treatment with antibiotics. The traveling bands demonstrated different features under normal growth conditions and for growth with antimicrobials, depending on the type of compound. Strong inhibition or bactericidal effects dominated when bacteria were subjected to antimicrobials close to the MIC, i.e., 2.5 mg/L for ampicillin, 0.015 mg/L for ciprofloxacin and 2.2 mg/L for gentamicin (Fig. S3). No dense transient bacterial migration peaks could be observed for these concentrations. In the case of 1.2 mg/L ampicillin, although the lag phase was nearly the same with and without antimicrobials, the traveling band peak appeared at *t* ≤ 4 h (Fig. 6d, orange curve), i.e., before ~7 h of the normal case (Fig. 6d, blue curve). For ciprofloxacin and gentamicin, the peak of the traveling band and the *v*_*mean*_ peak appeared nearly at the same time for all concentrations below the MIC (Fig. 8b, c), despite the confirmed growth inhibition effects of these compounds (Fig. S3e, f). This observation indicates that under antimicrobial treatment, there is no direct correlation between growth-related properties and motility features.

In principle, the presence of a chemical repellent can induce the phosphorylation of CheY and thus the clockwise activity of the flagellar motor, generating tumble events that drive bacteria to swim away from the chemical repellent^60,61^. However, not all chemicals that might be harmful to the bacteria can be considered repellents, which is the case for penicillin and streptomycin^62^. There is no clear evidence that ciprofloxacin and gentamicin act as chemorepellents for *E. coli*. For the ampicillin case, filamentation is the dominant effect, which strongly influences traveling band formation. However, for ciprofloxacin and gentamicin, we hypothesize that their concentrations have limited effects on *χ(s)* itself, which is confirmed by their similar TB distribution curves (Fig. 8h and Fig. S7h). Unlike for different inoculum sizes without drug treatment, the traveling band peak delay is not influenced by the lag phase elongation. In a previous study, we proposed that antimicrobials promote bacterial metabolic activity and induce enhanced energy spilling^63^. Higher energy consumption rates during bacterial growth also increase nutrient consumption dissolved in the culture medium. Consequently, the self-generated chemical gradient, represented by *f(b,s)* in Eq. 2, may develop more strongly and be formed on a shorter time scale. Here, the effect of *g(b,s)* and *h(b,s)* describing growth and death becomes less important. Our study proposes that the motility and collective behavior correlated with bacterial virulence might potentially be promoted by the presence of sub-MIC antimicrobials. We suggest that antimicrobial effects on metabolic activity represent the dominant factor influencing swimming behavior. This finding confirms the importance of correct antimicrobial prescriptions with appropriate doses in a therapeutic process. A more holistic quantitative study combining bacterial chemotaxis and metabolic effects under antimicrobial stress could reveal further interesting details in this respect.

Interestingly, the presence of traveling bands provides another method to perform fast AST for motile bacteria. The mean speed peak or cell density peak usually appears after 4 to 7 h (Figs. 6 and 8). Even if this read-out time is longer than the method based on single bacterial growth imaging, for instance^14^, MIC values can be safely determined on a much shorter time scale than with the gold standard broth microdilution method (typically 16 h to read-out)^30^. In the present proof-of-concept study, we performed AST starting from pure microbial colonies. AST starting from positive patient samples, including blood samples, for instance, might be feasible by implementing microfluidic toolboxes for sample preparation, purification and on-chip culture^64^. Moreover, high-content information assays based on different motility parameters may eventually open the path to optimized and personalized antimicrobial prescriptions. The proposed methodology may be adapted to various disease-relevant bacterial species other than *E. coli*, providing an interesting research approach that, eventually, can benefit clinical studies.

## Conclusion

Our microfluidic OA-based chip design provided specific advantages over other materials, enabling the fabrication of accurately defined shallow microfluidic channels for high-content imaging of individual bacterial trajectories in cell monolayers. By quantifying traveling band parameters in a quasi-2D plane, we revealed that isogeneic *E. coli* incubated from different inoculum sizes eventually developed phenotypic heterogeneity in swimming parameters. For instance, smaller inoculum sizes generated a higher degree of coordinated bacterial swimming behavior, appearing in the microchannel as traveling bands with high transient cell density and enhanced mean swimming speed. We propose that inoculum size may play the role of a noise source for gene expression, resulting in different manifestations of bistability. In our study, we focused on TB distribution as a direct phenotypic indicator and demonstrated the existence of low- and high-TB subgroups in migrating bacterial populations. The relative densities of low- and high-TB subpopulations in the traveling bands may explain the observed variations in cell density and swimming speed distributions. In particular, low-TB bacteria usually possess higher mean speeds and thus higher chemotactic ability associated with a higher effective diffusion coefficient. The impact of antimicrobial stress on the *E. coli* traveling bands depended strongly on the type of antibiotics and the concentration, especially in the case of ampicillin. For ciprofloxacin and gentamicin, the traveling bands were less affected or even revealed an enhancement of the chemotactic properties in specific cases. In the latter cases, traveling band peak delays were nearly independent of the antibiotic concentration, in contrast to the corresponding lag phase extensions of the growth curves. We suggest that these compounds enhance the bacterial metabolic activity and, as a consequence, the formation of the self-generated nutrient gradient in the microchannel, which is at the origin of the bacterial traveling band. This observation implies a possible compensation of opposing effects due to bacterial growth inhibition, nutrient depletion (metabolic activity) and chemotactic swimming, which is also reflected in the Keller-Segel model. Replacing the rich MH culture media with better controlled chemical environments, combined with theoretical simulations, could further unravel this question.

## Supplementary information

SupplementaryInformation_NoHighlight

Movie S1

## Data Availability

All relevant data needed to evaluate this work and the results in the paper are presented in the paper and in the Supplementary Information. Additional datasets, analysis details, and experimental parameters are available upon request.
