# Supplementary material for: Effect of inoculum size and antibiotics on bacterial traveling bands in a thin microchannel defined by optical adhesive
Source: Microsyst Nanoeng. 2021 Oct 22;7:86. doi: 10.1038/s41378-021-00309-3 (PMC8536744; doi:10.1038/s41378-021-00309-3)
Supplement: Supplementary file 1 — SupplementaryInformation_NoHighlight [file 41378_2021_309_MOESM1_ESM.docx]

**Supplementary Information**

Effect of Inoculum Size and Antibiotics on Bacterial Traveling Bands in a Thin Microchannel Defined by Optical Adhesive

Yang Liu, Thomas Lehnert and Martin A. M. Gijs*

Movie S1 An example of a video sequence showing bacteria and the swimming trajectories in a quasi-2D 4 μm high microfluidic channel (bacterial inoculum concentration 5×10^5^ CFU/mL, observation at 6 h, 40 f/s recorded for 10 s with a FOV of 340 μm × 340 μm).


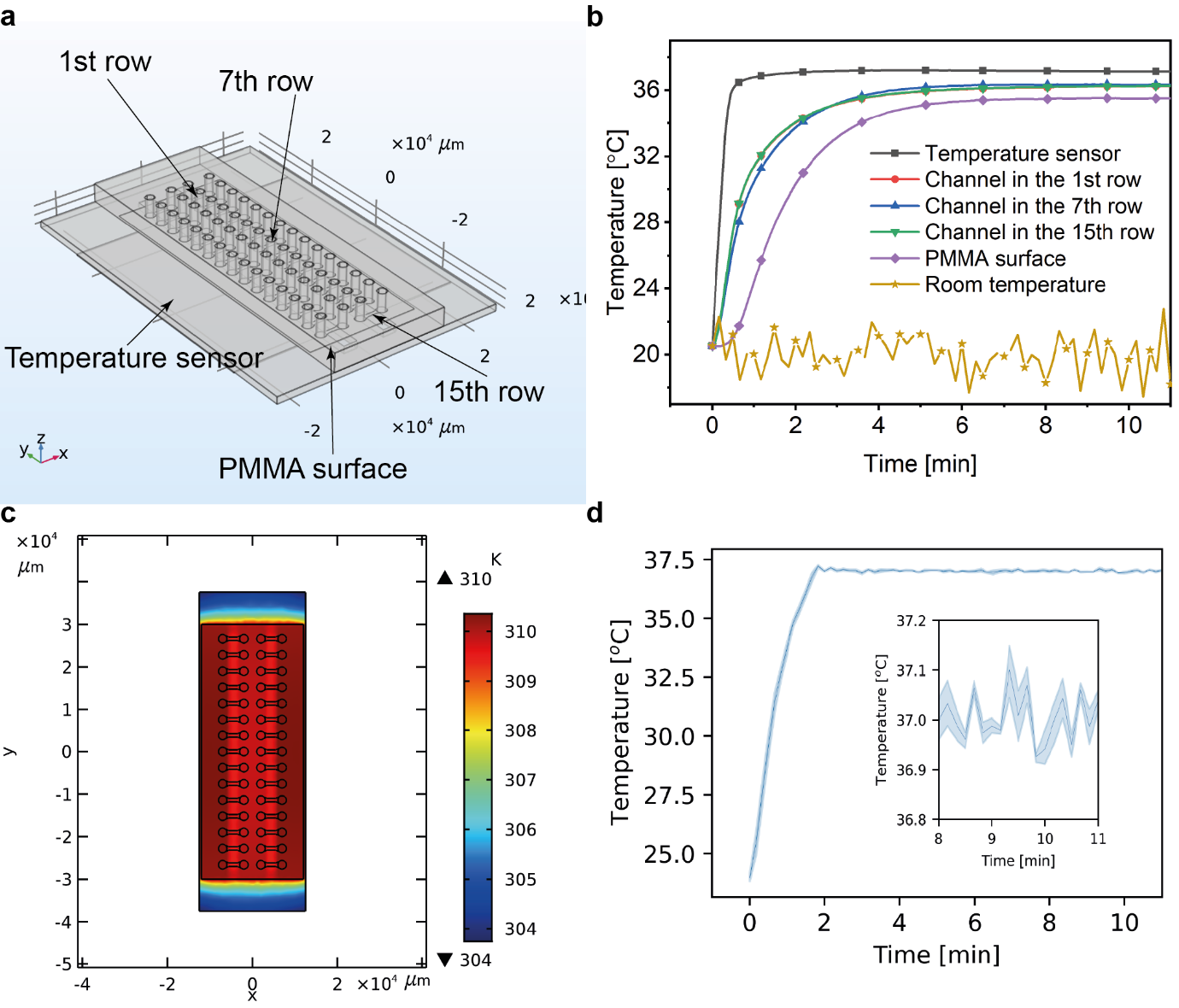


Fig. S1 Heat transfer simulation by COMSOL Multiphysics. (a) 3D model of the microfluidic chip (from top to bottom: PMMA, OA, and coverslip), Al plate and underlying heating pad. (b) Simulated temperature stabilization at different positions of the microfluidic chip, as indicated in (a): PID temperature sensor location, the channels in the 1^st^, 7^th^ and 15^th^ row, and the PMMA top surface, respectively. (c) Temperature distribution (simulation) of the microfluidic chips after heating for 10 min. The rectangular openings of the heating pads under each channel column result in a slight temperature inhomogeneity with a variation of ± 0.5 K. (d) Temperature measured by the RTD (Pt1000) sensor on the Al pad. The temperature stabilization at the sensor spot is in the range of ± 0.1K around the set point 37.0 ºC (inset in Fig. S2d). The first video acquisition for bacterial recording was done after 15 min to ensure temperature equilibration over the whole microfluidic chip.


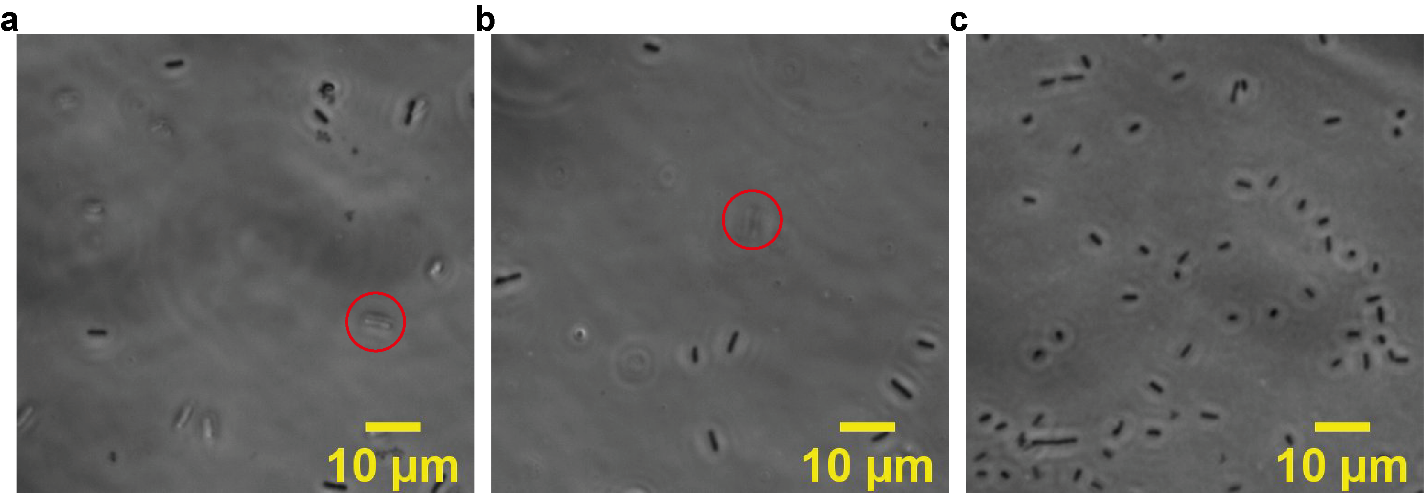


Fig. S2 Optimization of the channel height. (a) Height = 12 μm, (b) height = 8 μm, (c) height = 4 μm. The red circles indicate bacteria that are out of focus. All bacteria confined in a 4 μm high channel can be well focused on, thus this height was chosen for our microfluidic chip. Although a chip with a lower OA channel height can be fabricated, clogging at the inlet is likely to occur.


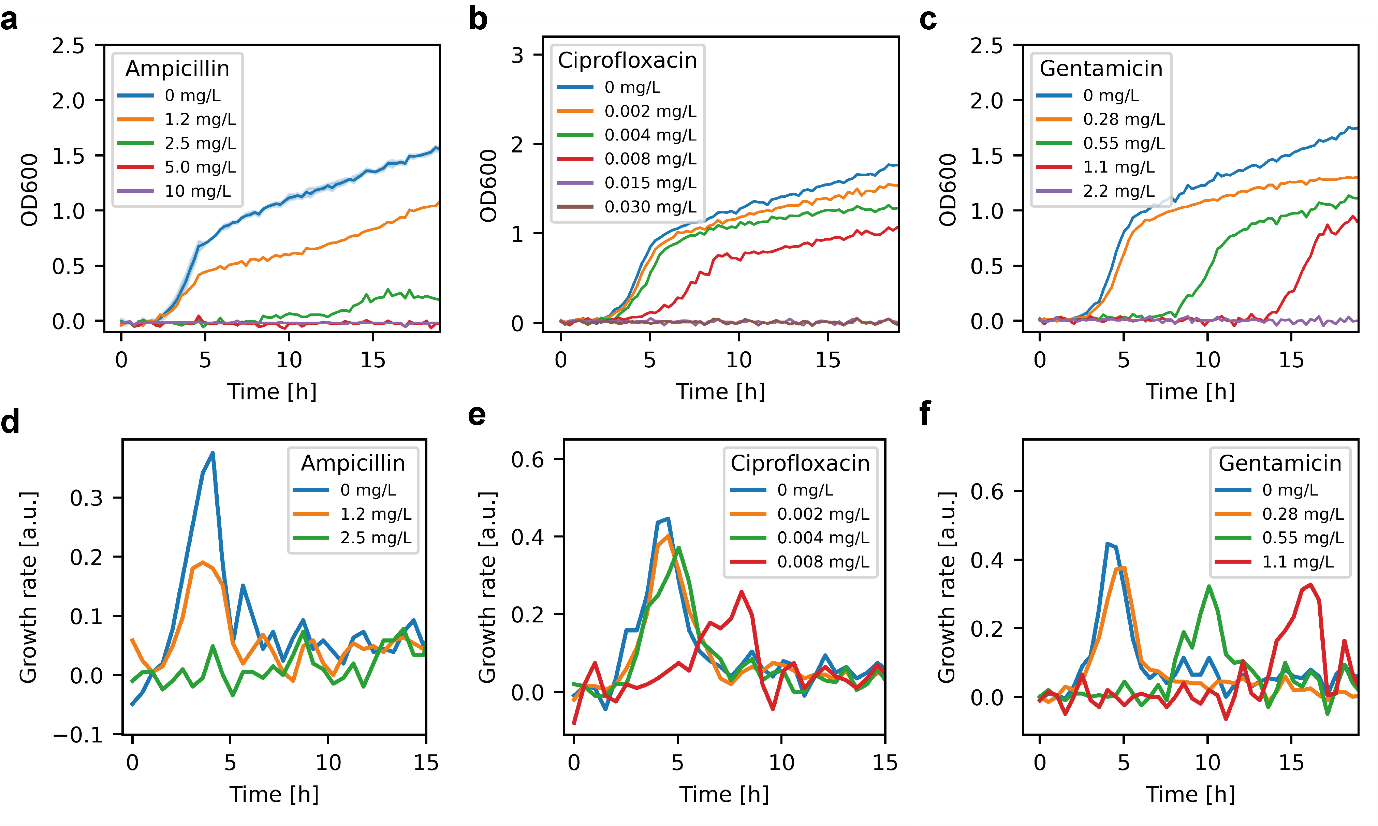


Fig. S3 *E. coli* growth and growth rate curves based on OD600 measurements as measured in a well plate. (a-c) Growth curves for *E. coli* exposed to different antibiotics: ampicillin (a), ciprofloxacin (b), gentamicin (c). (d-f) Corresponding growth rate curves (time derivatives of a-c): ampicillin (d), ciprofloxacin (e), gentamicin (f). Exposure to antimicrobials with concentrations approaching MIC results in strongly prolonged lag phases, *i.e.* for 2.5 mg/L ampicillin, 0.008 mg/L ciprofloxacin, 0.55 mg/L and 1.1 mg/L gentamicin, indicating progressive inhibition of bacterial growth. No growth can be seen above MIC. We determined the following MIC value ranges from curves in (a-c): (i) 2.5 < MIC ≤ 5 mg/L for ampicillin, (ii) 0.008 < MIC ≤ 0.015 mg/L for ciprofloxacin, and (iii) 1.1 < MIC ≤ 2.2 mg/L for gentamicin.


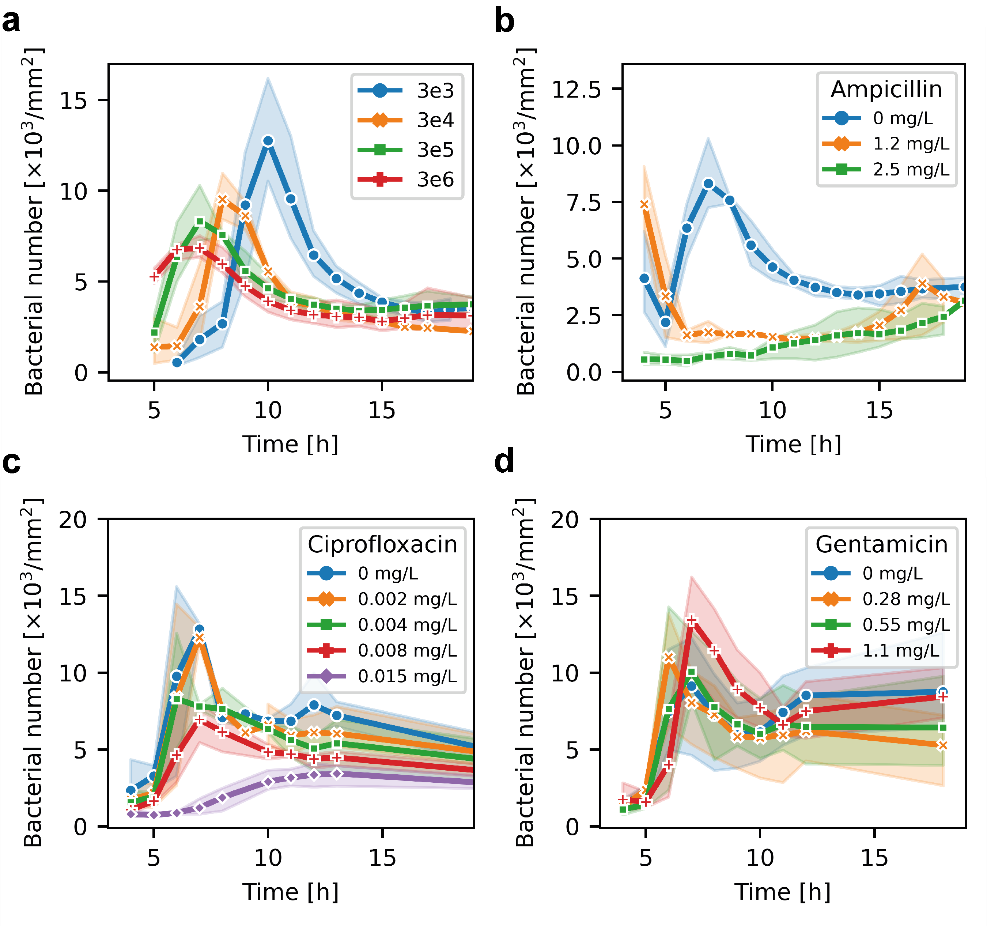


Fig. S4 Bacterial number density in the FOV as a function of time for different experimental conditions: inoculum size (a), ampicillin concentration (b), ciprofloxacin concentration (c), and gentamicin concentration (d). Bacterial number curves have the same overall shape as the cell density curves based on the integrated cell area measurements (Fig. 4), but due to filamentation stimulated by antibiotics, bacterial number curves do not reflect the true biomass. The error bars (or bands) stand for ± 2SE.


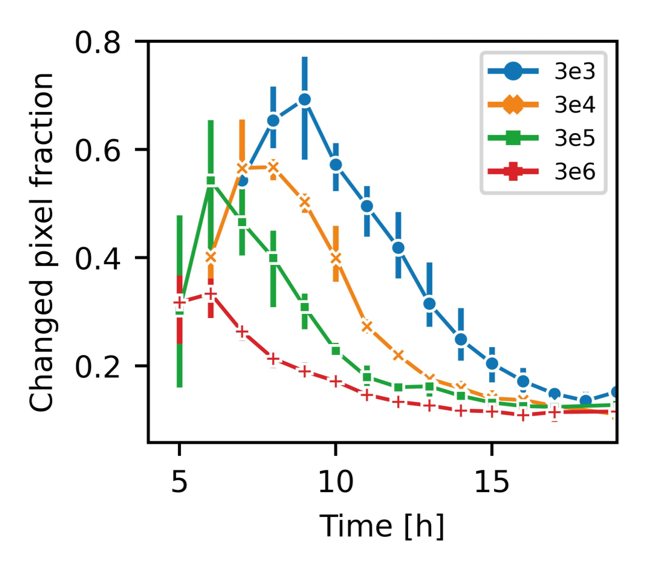


Fig. S5 Fraction of pixels changed between two consecutive frames averaged over the recorded image stacks in the FOV. A higher number of pixels changes indicates higher overall bacterial motility.


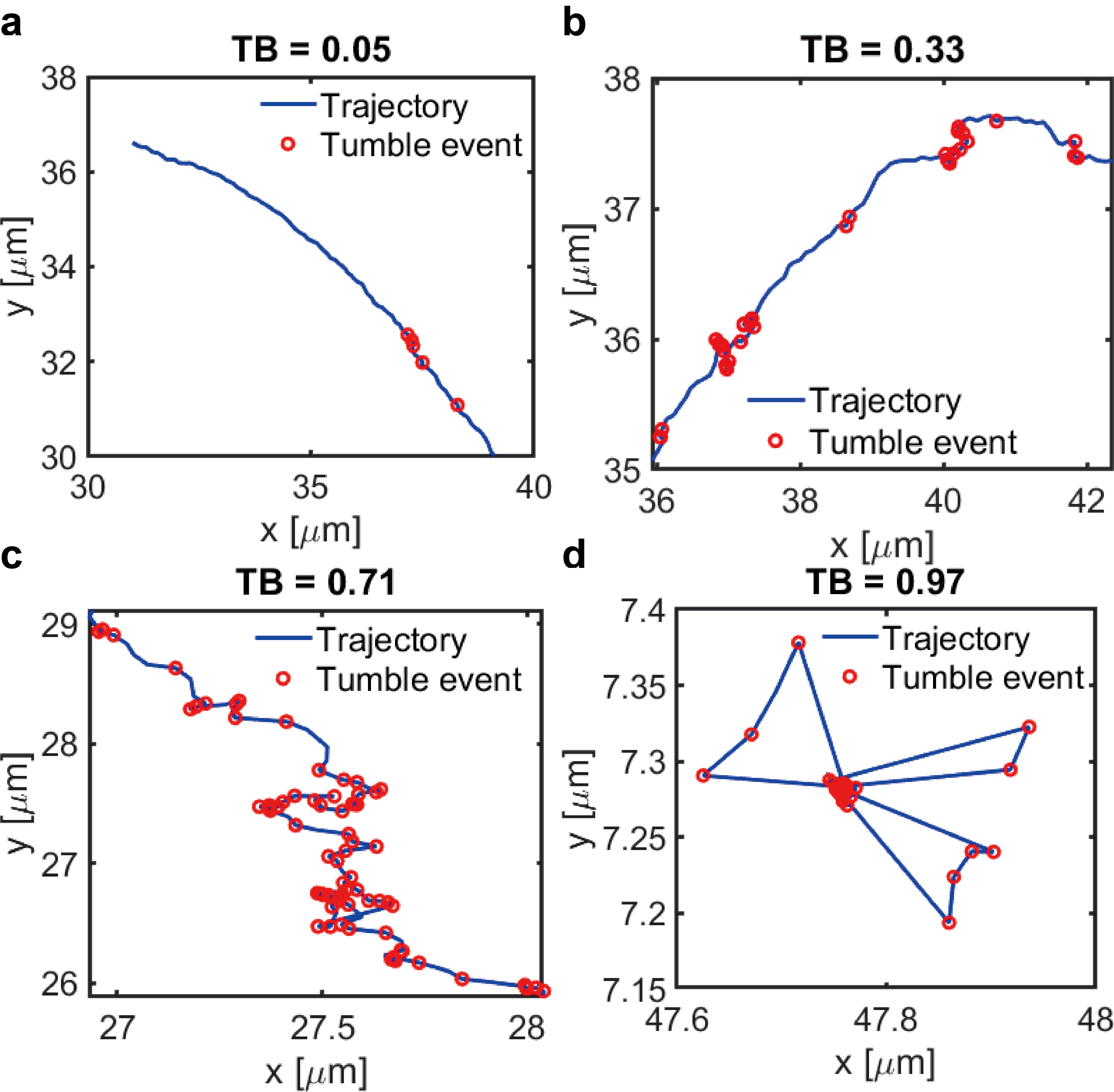


Fig. S6 Trajectories (blue lines) with different tumble bias (TB): (a) 0.05, (b) 0.33, (c) 0.71, and (d) 0.97. Red dots show the identified tumbling event. Low-TB trajectories have are more rectilinear, whereas high-TB trajectories indicate that bacteria change swimming directions more frequently. Bacteria with low TB tend to explore a larger area and have a higher swimming speed than the ones with high TB.


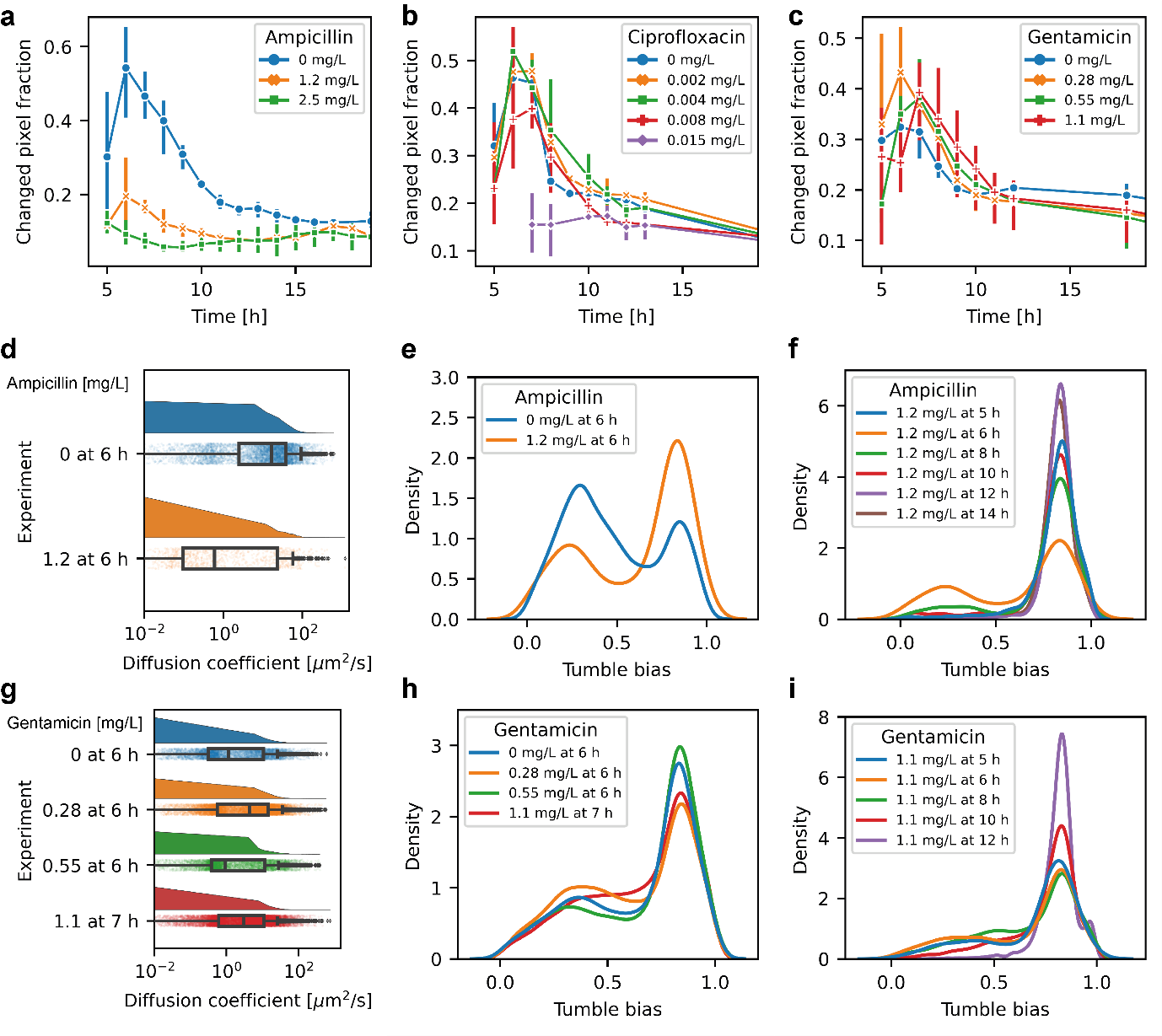


Fig. S7 (a-c) Pixel change analysis for bacteria in the different antimicrobials: ampicillin (a), ciprofloxacin (b), gentamicin (c). The error bars stand for ± 2SE. (d) *D_eff_* and (e) TB distribution for the time points with peak *v_mean_* for bacteria in 0 mg/L and 1.2 mg/L ampicillin. (f) TB distribution as a function of migration time for bacteria in 1.2 mg/L ampicillin. (g) *D_eff_* and (h) TB distribution of the time points with peak *v_mean_* for bacteria in 0, 0.28, 0.55 and 1.1 mg/L gentamicin. (i) TB distribution as a function of migration time for bacteria in 1.1 mg/L gentamicin.
